# Artificial Intelligence-Enhanced Multiparametric MRI and VI-RADS in Bladder Cancer: Current Evidence, Clinical Opportunities and Barriers to Translation

**DOI:** 10.3390/cancers18091322

**Published:** 2026-04-22

**Authors:** Cristian-Gabriel Popescu, Stefania Chipuc, Daniel Zgura, Bogdan Haineala, Anca Zgura

**Affiliations:** 1 Faculty of Medicine, Carol Davila University of Medicine and Pharmacy, 050474 Bucharest, Romania; 2 Faculty of Business and Tourism, Bucharest University of Economic Studies, 010374 Bucharest, Romania; 3 Department of Oncology-Radiotherapy, Alexandru Trestioreanu Institute of Oncology, 022328 Bucharest, Romania

**Keywords:** bladder cancer, multiparametric MRI, VI-RADS, artificial intelligence, radiomics, deep learning, muscle-invasive bladder cancer, response assessment, radiogenomics

## Abstract

Accurate distinction between non-muscle-invasive bladder cancer (NMIBC) and muscle-invasive bladder cancer (MIBC) remains the key local staging problem in bladder cancer because treatment intensity, timing of radical therapy, and suitability for bladder-preserving strategies all depend on it. Multiparametric magnetic resonance imaging (mpMRI) and the Vesical Imaging-Reporting and Data System (VI-RADS) now provide a standardized imaging framework for local staging and increasingly support MRI-first clinical pathways. Artificial intelligence (AI) has emerged as an additional decision-support layer, but the evidence base remains methodologically uneven. In this structured narrative review, we synthesized peer-reviewed literature from January 2020 to March 2026, while retaining foundational VI-RADS studies from 2018 to 2019, and prioritized guideline documents, meta-analyses, prospective cohorts, multicenter and externally validated AI studies, response-assessment studies, and papers addressing implementation and reporting quality. Current evidence shows that radiomics and deep learning models can achieve high discrimination for MIBC detection on MRI, and that the most plausible incremental value of AI lies in equivocal VI-RADS lesions, reader support outside high-volume expert settings, and multimodal risk stratification. However, most studies remain retrospective, highly selected, segmentation-dependent, and vulnerable to reference-standard bias, domain shift, and poor calibration. This review therefore emphasizes several translational issues that are often underreported: lesion-level versus patient-level inference, the distortive effect of TURBT-based labels, the need to evaluate false-negative consequences in VI-RADS 3 tumors, and the distinction between diagnostic support and broader pathway redesign. We also discuss response assessment, nacVI-RADS, segmentation automation, multicenter and federated infrastructure, workflow ownership, and the limits of imaging-only models in a biologically heterogeneous disease. The most credible near-term role of AI is not autonomous diagnosis, but augmentation of standardized mpMRI and VI-RADS within multidisciplinary care. Future progress will depend on prospective utility studies, site-held-out validation, transparent reporting, and the integration of imaging with molecular and cellular heterogeneity through radiogenomic and multi-omics approaches.

## Simple Summary

Bladder cancer treatment depends heavily on whether the tumor has invaded the bladder muscle. Standard tests sometimes leave uncertainty before major decisions such as radical cystectomy, bladder-preserving treatment, or repeat surgery. Multiparametric MRI and VI-RADS provide a structured way to estimate muscle invasion, but interpretation can vary and equivocal cases remain difficult. This review explains how artificial intelligence may support MRI interpretation by helping detect tumors, estimate the risk of muscle invasion, identify uncertain VI-RADS 3 lesions, and combine imaging with clinical or molecular information. Current evidence suggests that AI is most useful as a supervised second-reader or risk-stratification tool rather than as an autonomous decision maker. Important barriers remain, including small retrospective datasets, inconsistent imaging protocols, manual segmentation, imperfect pathology reference standards, limited external validation, and lack of proof that AI changes patient outcomes. Future work should prioritize prospective multicenter testing, calibration, decision-curve analysis, and integration into multidisciplinary workflows.

## 1. Introduction

Bladder cancer is the most common malignancy of the urinary tract and remains a major global cause of morbidity and mortality. In 2022, more than 600,000 new cases and more than 220,000 deaths were estimated worldwide, with a clear predominance in men [1]. Clinically, the most important early distinction is whether a tumor is confined to the mucosa and lamina propria or has invaded the muscularis propria. This binary staging boundary separates NMIBC from MIBC, but in practice it also separates very different treatment pathways, risk profiles, and timelines [2,3].

For NMIBC, management is centered on complete endoscopic resection, risk-adapted repeat transurethral resection, intravesical therapy, and surveillance. For MIBC, the clinical discussion shifts toward neoadjuvant cisplatin-based chemotherapy, radical cystectomy, perioperative systemic therapy, or carefully selected bladder-preserving trimodality treatment [2,3,4,5,6]. Understaging therefore risks delayed radical treatment and loss of a potentially curative window, whereas overstaging can expose a patient with organ-confined disease to unnecessary radical therapy and avoidable morbidity.

The conventional pathway of cystoscopy, transurethral resection of the bladder tumor (TURBT), and computed tomography is indispensable but imperfect for local staging. TURBT provides tissue diagnosis and remains central to management, yet it is susceptible to sampling error, incomplete representation of detrusor muscle, cautery artifact, tumor fragmentation, and mismatch between the resected specimen and the deepest invasive front visible on imaging [2,3]. Computed tomography remains important for upper tract and distant staging, but its soft-tissue contrast is limited for assessing the thin layered anatomy of the bladder wall [7,8].

The development of VI-RADS in 2018 was therefore a major advance. By integrating T2-weighted imaging (T2WI), diffusion-weighted imaging (DWI), and dynamic contrast-enhanced (DCE) imaging into a structured 5-point score, VI-RADS transformed bladder mpMRI from an occasional problem-solving tool into a standardized framework for local staging, communication, and increasingly, treatment planning [7,8,9,10]. Meta-analyses and multi-reader validation studies have since shown high diagnostic accuracy and generally good reproducibility, although performance remains most uncertain in the equivocal middle of the scale and in nonexpert settings [11,12,13,14,15].

At the same time, artificial intelligence has become a prominent theme in oncologic imaging. In bladder MRI, the most credible promise of AI is not to replace histology, radiologists, or make autonomous treatment decisions. Rather, AI may help standardize interpretation, generate continuous risk estimates around equivocal lesions, support MRI-first pathways, and connect local imaging with prognosis or response assessment [16,17,18]. This review was therefore revised to provide a more explicit and critical synthesis of how AI-enhanced mpMRI may contribute to bladder cancer care, where the current evidence is strongest, and which methodological and clinical barriers still prevent routine translation.

## 2. Scope and Structured Review Approach

This article is a structured narrative review rather than a systematic review or meta-analysis. The purpose was conceptual integration of a fast-moving translational field, not pooled effect estimation. To reduce subjectivity and selective citation, the review framework was made explicit and reproducible. PubMed/MEDLINE was searched iteratively between 1 January and 31 March 2026, and the search was supplemented by backward and forward citation reviews of key articles, guideline documents, and consensus statements. Search concepts combined terms for bladder cancer with terms related to mpMRI, VI-RADS, radiomics, machine learning, deep learning, transformer models, segmentation, response assessment, bladder preservation, radiogenomics, single-cell analysis, spatial transcriptomics, federated learning, and reporting methodology [7,16,17,18,19,20,21].

The primary eligibility window was January 2020 to March 2026 so that the review would cover at least the most recent five to six years of evidence, as requested by the reviewers. Foundational VI-RADS and early validation studies published in 2018–2019 were deliberately retained because they define the imaging substrate on which later AI work depends [7,22,23]. Priority was given to: (1) EAU and other major guidance documents; (2) meta-analyses and systematic reviews; (3) prospective studies; (4) multicenter or external-validation AI studies; (5) clinically oriented response-assessment studies; (6) public dataset or federated-learning resources; (7) papers addressing reporting quality, implementation, and translational methodology [2,3,16,17,20,21,24,25,26].

Studies were de-emphasized when they were very small proof-of-concept reports without a clinically interpretable endpoint, duplicate analyses of overlapping cohorts, or purely technical papers with no direct relevance to muscle invasion, response assessment, segmentation workflow, or clinical implementation. When multiple publications arose from the same research group or partially overlapping datasets, the more comparative, externally validated, or clinically mature study was preferentially emphasized. The synthesis was then organized around the questions most relevant for translation: how mpMRI and VI-RADS currently perform; what different classes of AI add beyond expert human interpretation; where bias most commonly enters the literature; what evidence is strongest in equivocal lesions and response assessment; how segmentation, multicenter validation, and workflow ownership affect implementation; why future models should be linked to molecular heterogeneity rather than imaging alone.

This approach does not claim exhaustive capture of every engineering study. It does, however, make the logic of evidence selection explicit and aligns the review more closely with the needs of clinicians, radiologists, methodologists, and translational investigators evaluating whether AI-enhanced mpMRI is ready to alter patient management. The resulting framework is summarized in Table 1.

## 3. Why Accurate Preoperative Staging Remains an Unmet Need

The central preoperative question in newly diagnosed bladder cancer is deceptively simple: has the tumor invaded the muscularis propria? In practice, the answer is often uncertain because bladder tumors can be exophytic, broad-based, multifocal, inflamed, partially resected, or obscured by hemorrhage and edema. The relevant anatomic interface is thin, dynamic, and highly sensitive to bladder distension. These challenges make local staging fundamentally different from detecting large solid-organ masses [7,8,19].

TURBT is essential but not a perfect depth-of-invasion assay. The deepest invasive front may not be sampled, detrusor muscle may be absent, cautery may obscure key landmarks, and in multifocal disease the imaged lesion, the resected lesion, and the final dominant pathology specimen may not align perfectly [2,3,23]. This matters because incorrect local staging propagates immediately into inappropriate timing of repeat resection, premature reassurance, or delayed referral for neoadjuvant therapy, cystectomy, or bladder-preserving chemoradiation.

The clinical importance of preoperative staging became especially tangible in the BladderPath trial, in which an MRI-based pathway shortened time to correct treatment for patients with suspected MIBC [24]. The lesson is not that histology becomes obsolete; it does not. Rather, a sufficiently reliable imaging pathway can reshape the sequence and urgency of invasive procedures. This is the context in which both VI-RADS and AI matter most: not as abstract image-analysis exercises, but as tools that may change when and how the right treatment is initiated.

## 4. mpMRI and VI-RADS as the Imaging Substrate

mpMRI combines complementary anatomic and functional information. T2WI depicts bladder wall architecture, stalk configuration, and continuity of the low-signal muscular layer. DWI and the apparent diffusion coefficient (ADC) map provide information on cellularity and diffusion restriction, often sharpening suspicion of deeper invasion. DCE imaging can help distinguish an enhancing tumor from submucosal stalk, edema, or inflammatory change when other sequences are equivocal [7,8,9,10]. No single sequence solves the staging problem on its own; the value lies in the structured integration of all three.

VI-RADS operationalizes that integration in a reproducible 5-point score. Scores 1–2 indicate that muscle invasion is unlikely, score 3 remains equivocal, and scores 4–5 indicate likely or very likely muscle invasion [7,10]. The score has gained acceptance because it provides more than discrimination alone: it creates a common language across radiology, urology, medical oncology, and radiation oncology, making mpMRI usable in multidisciplinary practice rather than as an isolated radiology opinion [8,19].

The evidence supporting VI-RADS is now relatively mature compared with most bladder MRI-AI literature. Meta-analyses consistently show strong pooled sensitivity and specificity, and interobserver reliability is generally good, particularly in experienced hands [11,12,13,14,15]. At the same time, several caveats remain highly relevant for AI development: image quality is fragile; the score is less stable in the VI-RADS 3 gray zone; post-biopsy timing influences interpretation; expertise still matters. AI therefore needs VI-RADS not as a competitor, but as a high-quality clinical scaffold.

Table 2 summarizes the practical role of each mpMRI component, the sequence-specific pitfalls most likely to cause human and algorithmic error, and where published evidence for AI integration is strongest versus still aspirational.

## 5. What AI Adds Beyond Visual Interpretation

The phrase “AI in bladder MRI” often groups together methods that are technically and clinically quite different. Radiomics usually begins with a segmented lesion and extracts handcrafted intensity, texture, shape, or wavelet features before applying a conventional classifier such as logistic regression, support vector machines, or random forests [27,28,29]. This approach can work in smaller datasets and may be more transparent at the feature-engineering stage, but it is heavily dependent on segmentation, preprocessing choices, and harmonization assumptions.

Deep learning models, most commonly convolutional neural networks, learn hierarchical features directly from images and can theoretically capture both lesion texture and contextual anatomy without predefined features [16,30,31,32]. Transformer-based models extend this idea through self-attention and broader contextual modeling, but in bladder MRI they remain early, data-hungry, and not yet clearly superior to well-designed convolutional or hybrid architectures [33]. Multimodal fusion systems go one step further by combining imaging with morphology, clinical variables, pathology, or even transcriptomic data; conceptually, they are attractive for prognostication, but they are also the least mature and the most vulnerable to hidden confounding [34,35,36].

From a clinical perspective, these categories should not be discussed as if they were interchangeable. Handcrafted radiomics and conventional machine learning are the most mature in terms of small-sample feasibility, but they are also the most sensitive to contouring and image standardization. Reader-support deep learning systems are closer to plausible near-term deployment for muscle invasion detection, especially when layered on top of VI-RADS. Transformer models and radiogenomic fusion systems are currently better viewed as exploratory rather than practice ready. This relative maturity is important because many review articles mention the methods together without making clear which tasks are realistic now and which remain aspirational [16,17,37].

Equally important, AI does not compensate for poor imaging. Inadequate bladder filling, motion, recent instrumentation, post-biopsy hemorrhage, and sequence heterogeneity degrade both human and algorithmic performance [7,8,19]. The most clinically plausible model is therefore layered rather than disruptive: standardized pretreatment mpMRI, structured VI-RADS interpretation, and AI used as a calibrated second reader or risk-enrichment module rather than as an autonomous diagnostic endpoint.

## 6. Current Evidence for MRI-Based AI in Bladder Cancer

### 6.1. Detection of Muscle Invasion

The strongest AI literature in bladder MRI concerns discrimination between NMIBC and MIBC. Across meta-analyses, pooled performance is usually high, with overall AUCs around 0.9, but the headline numbers conceal important heterogeneity [16,17]. The 2024 review by He et al. found pooled AUCs of 0.92 for MRI-based AI models, 0.91 for deep learning, and 0.89 for radiomics, while also concluding that methodological and reporting quality were generally poor and that risk of bias was high across the included studies [16]. The larger 2025 meta-analysis by Wang et al. reached a similar pooled AUC of 0.92, with pooled sensitivity and specificity of 0.86 each, but identified substantial heterogeneity according to center type, validation strategy, segmentation method, and geographic origin [17]. This is not a trivial caveat: it means that apparently similar AUCs can arise from very different development pathways and very different prospects for real-world transportability.

A comparison across study designs is therefore more informative than citing global discrimination alone. Early radiomics work demonstrated proof of concept, but systematic reviews consistently found strong dependence on manual segmentation, small retrospective cohorts, and poor reporting of feature robustness or harmonization [27,28,29]. Newer deep learning studies are more likely to include external testing, yet many still rely on curated lesions rather than end-to-end patient-level workflows. For example, Li et al. trained a T2WI-based deep learning model and reported AUCs of 0.963 internally and 0.861 externally, with the most relevant gain occurring in VI-RADS 2–3 tumors rather than in clearly low- or high-risk cases [30]. In a subsequent comparison, the same group showed better external discrimination for multi-task deep learning than for radiomics or single-task deep learning, suggesting that architecture and training objective matter, not just the imaging modality [31].

Later multicenter studies broadened the architectural landscape but also highlighted the persistence of generalization problems. Kurata et al. reported that a vision-transformer model achieved performance comparable to junior radiologists when regions of interest were manually defined, but performance dropped when the segmentation step was less controlled [33]. Cai et al. likewise reported strong internal performance for a multicenter T2WI-based deep learning model, yet external accuracy still declined markedly relative to development data [32]. These studies support the view that the current question is no longer whether AI can learn the task under favorable conditions. The real question is whether the full pipeline—image acquisition, localization, classification, uncertainty estimation, and clinical interpretation—remains reliable across institutions, scanners, and patient populations.

A fair comparative conclusion is therefore nuanced. AI can match or sometimes outperform expert interpretation in selected cohorts, especially around equivocal cases or in readers with less bladder MRI experience. However, the situations in which AI clearly surpasses experienced VI-RADS readers remain relatively narrow. For most current datasets, expert VI-RADS remains a strong benchmark, and claims of superiority should be interpreted cautiously unless the study includes external validation, clinically meaningful thresholds, and explicit comparison with expert rather than junior readers [16,17,30,33].

### 6.2. Equivocal VI-RADS Lesions, Calibration, and Decision-Relevant Evaluation

The most important translational use case is not the easy tumor but the equivocal one. VI-RADS 3 lesions sit exactly where management uncertainty is greatest: they may represent true early muscle invasion, bulky T1 disease, inflammation, or post-procedural change. An AI tool that improves this gray zone could be clinically meaningful even if it produces only modest gains in overall AUC. Several studies have therefore combined VI-RADS with additional quantitative information such as radiomics signatures, tumor contact length, or nomograms focused specifically on VI-RADS 3 disease [38,39,40,41].

This setting also exposes why discrimination alone is insufficient. A clinically useful model for VI-RADS 3 lesions should be evaluated against decision-relevant endpoints: the false-negative rate for MIBC; the negative predictive value required to defer radical escalation; the number of repeat TURBTs potentially avoided; the number of patients expedited to neoadjuvant therapy or bladder-preservation workup; the downstream consequences of incorrect reclassification. Missing a small number of truly muscle-invasive tumors may be far more harmful than overcalling a subset of aggressive T1 lesions. Accordingly, future studies should report calibration, predefined thresholds, and decision-curve utility rather than only sensitivity, specificity, or AUC [20,21].

The unit of evaluation matters here as well. A lesion-level model may look impressive if tested on manually cropped tumors, but the patient-level question is different: does the model improve the treatment recommendation for the person sitting in the multidisciplinary meeting? A clinically relevant benchmark in score-3 lesions should therefore compare standard VI-RADS reporting against AI-augmented reporting in terms of consensus confidence, MDT decisions, false-negative consequences, and pathway timing. In other words, the benchmark should move from “Can the algorithm classify the lesion?” to “Did it change the right decision for the right patient?”.

### 6.3. Prognostication and Multimodal Fusion

Imaging also invites a broader question: can AI extract information that is biologically or prognostically useful beyond binary local staging? Multimodal fusion models are the most visible attempt to answer that question. In a large multicenter retrospective study, Cai et al. combined deep learning features, radiomics, morphology, and clinical variables and reported better overall survival discrimination than pathologic T stage alone [34]. Proof-of-concept radiogenomic studies have further suggested that MRI or combined CT/MRI features may capture aspects of transcriptomic structure relevant to aggressive behavior [35,36].

These results are conceptually important, but they should not yet be overinterpreted. Survival modeling in retrospective multicenter datasets is vulnerable to confounding by treatment selection, stage migration, center effects, and hidden correlations between imaging quality and care quality. A model can therefore be statistically interesting without being clinically actionable. At present, prognostic AI in bladder MRI is better viewed as hypothesis-generating and potentially trial-enriching than as a ready-made tool for individual treatment intensification.

### 6.4. Response Assessment, Neoadjuvant Therapy, and Bladder Preservation

Response assessment after systemic therapy is one of the most promising but still early extensions of bladder mpMRI. nacVI-RADS and related post-treatment MRI frameworks provide a structured language for assessing response after neoadjuvant chemotherapy or immunotherapy [18,42,43,44,45]. In a prospective validation study, Dehghanpour et al. reported good diagnostic accuracy and excellent inter-reader agreement for detecting complete response after neoadjuvant chemotherapy [43]. Additional series in pembrolizumab-treated cohorts linked post-treatment VI-RADS or nacVI-RADS assessments with pathologic downstaging and oncologic outcomes [44,45].

The strategic importance of this area is clear. If response-adapted bladder preservation becomes more common, clinicians will need imaging tools that estimate residual viable disease with enough confidence to support organ-preserving decisions and early salvage when needed [4,5,6]. However, the evidence base is still modest, the cohorts are highly selected, and organ-preservation decisions cannot currently rest on MRI or AI alone. The field remains at the stage of structured feasibility and early validation rather than definitive response-adapted treatment selection.

This distinction should remain explicit in review writing. AI-based response assessment is timely and potentially high impact, but it is not yet mature enough to be discussed in the same evidentiary register as baseline VI-RADS staging. The correct framing is that mpMRI and nacVI-RADS provide a necessary substrate for future response-adapted models, not that they have already solved the problem of safe organ preservation.

## 7. Critical Appraisal of the Evidence Base

A major weakness of the literature is that methodological limitations are often acknowledged only in general terms. It is not enough to state that most studies are retrospective and at high risk of bias; the common biases should be named because they affect reported performance in predictable ways. First, spectrum and selection bias are pervasive. Many cohorts come from tertiary centers, include only patients with good-quality pretreatment MRI and complete pathology, and exclude precisely the difficult real-world situations—catheterization, heavy hematuria, poor distension, urgent bleeding, incomplete protocols, frailty, or recent instrumentation—in which staging is most challenging [16,17,29]. Such selection usually inflates apparent accuracy.

Second, reference-standard bias is central in bladder MRI and deserves more emphasis than it usually receives. TURBT pathology is often used as the ground truth because it is available in most patients, but it is itself an imperfect measure of depth of invasion [2,3]. When the reference standard can understage disease, model training is distorted and validation becomes difficult to interpret. Cystectomy pathology is anatomically more robust for final T stage, but it is available only in a selected subset and may be separated from imaging by interval treatment. Some response or pathway studies use longitudinal outcomes rather than direct histopathology, which may be clinically relevant but less precise for model supervision. The implication is that “ground truth” in bladder MRI is often conditional, and studies should state explicitly which pathological or clinical label they are using and what kind of misclassification it may introduce.

Third, the distinction between lesion-level, tumor-level, and patient-level inference is frequently blurred. Lesion-level inference asks whether a cropped or segmented lesion is muscle invasive. Tumor-level inference is similar but presumes that the lesion under analysis corresponds to the dominant tumor. Patient-level inference is different: it asks whether the patient should enter an NMIBC-oriented or MIBC-oriented pathway. These levels are not interchangeable. A lesion-level AUC from manually delineated regions of interest may overestimate patient-level utility because it assumes lesion detection is already solved, that the correct lesion was selected, and that multifocal disease can be reduced to one target. Review articles should therefore interpret performance metrics through the lens of the unit of analysis rather than as if all AUCs addressed the same clinical question.

Fourth, annotation and segmentation bias remain substantial. Many high-performing models depend on expert manual contours, which are costly, difficult to scale, and partly circular if the contour itself is informed by the same imaging features that influence the stage label [30,31,46,47]. Fifth, leakage and validation bias are often underexplored. Random train-test splits within the same institution, repeated scans from related acquisition settings, or lesion-based splits from the same patient can all produce optimistic estimates. Site-held-out testing and temporally separated validation are still uncommon relative to the claims made.

Finally, discrimination metrics dominate the field, whereas calibration and utility analyses remain sparse. A model with a strong AUC may still be clinically unsafe if it systematically overestimates or underestimates risk at the thresholds that matter for treatment. For translation, bladder MRI-AI studies should routinely report calibration plots, Brier-type measures, uncertainty intervals, clinically meaningful threshold analyses, and decision-curve methods, and should align their reporting with CLAIM, TRIPOD + AI, and STARD-AI principles [20,21,26].

## 8. Multicenter Validation, Segmentation Burden, and Federated Learning

Generalizability depends not only on model architecture but also on infrastructure. Bladder MRI differs from many other imaging AI domains because the target anatomy is a thin wall whose appearance changes with distension, motion, intraluminal content, recent biopsy, and protocol details. Small differences in sequence parameters or timing can therefore shift the visual boundary that the model is asked to learn [7,8,19]. This partly explains why domain shift is so prominent in the field and why simple transfer of a model from one institution to another is unreliable.

Segmentation is a particularly important bottleneck. Manual segmentation remains common and often provides the best apparent performance because the classifier receives a carefully curated lesion mask. Semi-automated approaches reduce labor but may still require user intervention or quality control. Fully automated pipelines are most scalable, yet they introduce error propagation because the system must both localize the lesion and classify invasion [46,47,48,49]. The available literature suggests that some performance degradation is typical as automation increases; the drop reported in transformer and segmentation studies is therefore not a minor technical inconvenience but a central translational issue [33,46,47].

Publicly available multicenter data are beginning to address these limitations. The dataset released by Cao et al. is particularly important because it provides three-dimensional T2WI scans from four centers with invasion labels and pixel-level annotations, and it also established federated-learning baselines for classification and segmentation [25]. Federated or privacy-preserving learning is attractive in bladder MRI because many institutions have modest case volumes, data sharing is sensitive, and heterogeneity itself must be learned rather than suppressed. However, data availability alone is not enough. Transportable models will require site-held-out testing, temporal validation, subgroup analysis, explicit failure review, and workflow-aware evaluation rather than only retrospective benchmark comparisons.

Table 3 provides a comparative summary of representative MRI-based AI studies and resources, with specific attention to model class, validation strategy, reference standard, inference level, and segmentation burden.

## 9. Clinical Translation: Diagnostic Support Versus Pathway Redesign

Potential clinical impact should be discussed in two distinct categories that are often conflated. Diagnostic support means improving local staging confidence and consistency within the existing pathway. Pathway redesign is different: it means changing the sequence of investigations, accelerating correct treatment, reducing unnecessary repeat TURBT, or altering entry into neoadjuvant and bladder-preserving strategies. High AUC for muscle invasion mainly supports the first claim; the second requires prospective pathway or implementation evidence rather than retrospective classification benchmarks alone [19,24].

**Figure 1:**
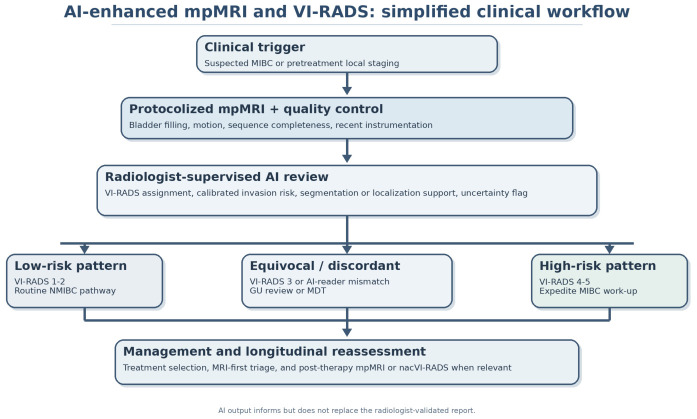
Simplified clinical workflow for AI-enhanced mpMRI and VI-RADS. AI is positioned as a radiologist-supervised decision-support layer that informs, but does not replace, staging and reassessment decisions. Arrows indicate workflow progression and feedback loops between image acquisition, radiologist interpretation, multidisciplinary decision-making, and treatment reassessment.

In a pragmatic MRI-first model, AI would usually run after a protocolized pretreatment bladder mpMRI and return three radiologist-facing outputs: image-quality or lesion-localization flags, a calibrated probability of muscle invasion, and an uncertainty marker for VI-RADS 3 or radiologist-AI discordant cases. The radiologist remains accountable for the final report, and urologists or oncologists should act on the radiologist-validated interpretation rather than on raw algorithmic output. Figure 1 presents this workflow in simplified form.

The figure therefore emphasizes ownership and escalation points rather than autonomous decision-making.

## 10. Remaining Implementation Barriers

Several implementation barriers remain decisive. Domain shift is the most obvious. Bladder filling, motion, field strength, coil configuration, sequence timing, and interval from biopsy or TURBT can alter the visual appearance of the bladder wall enough to destabilize both human and algorithmic interpretation [7,8,16,17]. Unlike AI tasks centered on large parenchymal masses, bladder MRI depends on thin interfaces and subtle layer disruption; harmonization is therefore unusually difficult and cannot be treated as a minor technical afterthought.

A second barrier is deployment infrastructure. Hospitals need interoperable software, quality-control gates, audit trails, and governance within radiology leadership, not just a high-performing classifier. The operational questions are practical but crucial: how does the tool enter the reporting workflow, how are failures logged, and what escalation pathway exists when the algorithm and radiologist materially disagree?

Interpretability also requires a more critical discussion than it often receives. Heatmaps, saliency maps, or attention overlays can indicate where a network is focusing, but they do not necessarily constitute a clinically meaningful explanation. They rarely answer the question clinicians actually ask: which anatomical feature or biologically plausible pattern justifies changing management? In high-stakes decisions such as cystectomy referral or organ-preservation selection, saliency should therefore be treated as supportive visualization rather than as proof of trustworthy reasoning.

Finally, prospective utility and cost-effectiveness evidence remain limited. Regulators, hospitals, and clinicians will ultimately ask whether AI-enhanced mpMRI changes management, avoids unnecessary procedures, shortens time to correct treatment, or improves outcomes at acceptable cost. Until that level of evidence exists, implementation will remain cautious even for technically strong models. Even if those operational hurdles are solved, however, imaging-only systems would still face a separate ceiling: they capture anatomy more readily than tumor biology. These implementation barriers and the corresponding mitigation strategies are summarized in Table 4.

## 11. Molecular Heterogeneity, Radiogenomics, and the Limits of Imaging-Only Models

This review focuses primarily on AI-enhanced mpMRI, and that focus has an important limitation: imaging-only models cannot fully represent the molecular, cellular, and clonal heterogeneity of bladder cancer. Urothelial carcinoma is biologically diverse across luminal and basal programs, stromal composition, immune infiltration, treatment pressure, and clonal evolution. Even when a model predicts muscle invasion accurately, it may still miss the biological features that drive response to chemotherapy, immunotherapy, radiotherapy, or early metastatic spread [35,36,50,51,52,53].

Recent bladder-specific single-cell and spatial studies show why this matters. Single-cell RNA sequencing has identified inflammatory fibroblast states, immune-contexture differences, and cell-state variation between primary and recurrent lesions that are invisible to routine staging MRI [50,51,52]. Spatial and multi-omic analyses further suggest that microenvironmental organization, lineage programs, and clonal evolution may influence recurrence and treatment response [53,54]. Similar integrative frameworks in melanoma and hepatocellular carcinoma reinforce the broader principle that imaging becomes more informative when it is linked to cellular and immune-state heterogeneity [55,56].

Accordingly, future radiogenomic programs should pair pretreatment mpMRI with TURBT or cystectomy tissue, bulk RNA sequencing, single-cell or spatial transcriptomics, and, where feasible, circulating biomarkers. The aim is not omics for their own sake, but risk models that capture not only muscle invasion, but also biologically meaningful states relevant to systemic therapy, radiotherapy, and organ preservation [35,36,50,53,54].

## 12. Future Directions

The next phase of the field should move from isolated retrospective classifiers to prospectively evaluated clinical systems. Future studies should predefine the intended use case—reader support, equivocal-lesion triage, response assessment, or prognostic enrichment—and should evaluate lesion-level, patient-level, and pathway-level endpoints separately. Calibration, uncertainty, subgroup performance, and failure analysis should be reported alongside discrimination metrics in line with modern AI reporting standards [20,21,26].

Methodologically, that means site-held-out and temporal validation, stronger pathology linkage, and explicit analysis of how performance changes as segmentation becomes more automated [25,46,47,49]. Clinically, it means pathway-based trials asking whether AI-enhanced mpMRI reduces time to correct treatment, improves management of equivocal disease, or supports safer response-adapted bladder preservation [18,24,43]. Biologically, it means linking imaging to radiogenomics and multi-omics so that risk estimates reflect more than anatomy alone [35,36,50,54].

The systems most likely to matter are not simply the most accurate in curated datasets, but the ones that remain stable across centers and improve real decisions in difficult cases.

## 13. Conclusions

AI-enhanced mpMRI is a promising extension of bladder cancer imaging, but the current evidence supports a specific—not unlimited—clinical claim. Standardized mpMRI and VI-RADS are already useful for local staging, and AI appears most credible as a calibrated second-reader layer for equivocal lesions, response-assessment research, and multimodal risk stratification [7,11,12,16,17].

Routine adoption now depends less on producing additional retrospective classifiers and more on delivering systems that are externally validated, calibrated, workflow-compatible, and biologically better contextualized. If prospective studies show that such systems improve treatment selection and timing, AI-enhanced bladder mpMRI could become a practical component of multidisciplinary precision care.

## Figures and Tables

**Table 1:** Structured narrative-review framework used to guide literature selection and synthesis.

Element	Approach
Review type	Structured narrative review focused on clinically relevant evidence rather than pooled meta-analysis.
Primary source	PubMed/MEDLINE, supplemented by backward and forward citation reviews of key papers, guidelines, and consensus statements.
Time window	January 2020 to March 2026, with foundational VI-RADS papers from 2018 to 2019 intentionally retained.
Core search domains	Bladder cancer; mpMRI; VI-RADS; radiomics; machine learning; deep learning; transformers; segmentation; response assessment; bladder preservation; radiogenomics; single-cell or spatial omics; federated learning; reporting methodology.
Prioritized evidence	Guidelines; meta-analyses; prospective cohorts; multicenter or external-validation AI studies; response-assessment studies; public datasets; papers on reporting, calibration, and implementation.
De-emphasized evidence	Very small proof-of-concept studies without clinical endpoints, duplicate analyses of overlapping cohorts, and purely technical papers without direct staging or translational relevance.
Evidence weighting	Higher weight given to external validation, prospective design, clinically interpretable endpoints, and pathway relevance.
Main synthesis domains	Imaging substrate; AI categories; muscle invasion detection; equivocal VI-RADS lesions; response assessment; prognostication; segmentation and infrastructure; implementation; multi-omics integration.

**Table 2:** Clinical role of mpMRI components and where AI integration is already supported versus still aspirational.

Component	Main Clinical Role	Contribution to Muscle Invasion Assessment	Frequent Limitations	AI Integration: Current Evidence vs. Aspirational Use
T2WI	High-resolution anatomy, lesion morphology, stalk assessment, and appraisal of bladder wall continuity.	Dominant sequence for lower VI-RADS categories and structural evaluation of muscular-layer interruption.	Suboptimal distension, motion, clot, post-TURBT change, and dependence on reader experience.	Evidence now: morphology extraction, ROI-based classification, lesion localization. Aspirational: fully automated wall analysis in routine MRI-first pathways.
DWI/ADC	Functional assessment of cellularity and diffusion restriction.	Particularly informative when deeper invasion is suspected and when T2WI findings are borderline.	Susceptibility artifact, distortion, lower signal-to-noise ratio, and variable b-values.	Evidence now: radiomics, voxel-wise invasion probability, response monitoring. Aspirational: robust cross-scanner harmonized quantitative maps.
DCE	Assessment of enhancement kinetics and distinction between tumor, stalk, and inflammatory change.	Helpful when DWI or T2WI is equivocal, especially in intermediate-risk lesions.	Contrast-timing variability, protocol inconsistency, gadolinium use, and motion.	Evidence now: multimodal fusion in selected cohorts. Aspirational: standardized temporal modeling for routine clinical deployment.
Integrated VI-RADS score	Structured 1–5 probability score communicating local stage across the MDT.	Translates mpMRI into a clinically actionable estimate of muscle invasion.	Score-3 gray zone, training requirements, site-level implementation variability.	Evidence now: AI as second reader, combined radiomics + VI-RADS models, equivocal-lesion risk refinement. Aspirational: prospectively validated threshold-based management support.

**Table 3:** Comparative summary of representative MRI-based AI studies and data resources in bladder cancer.

Study	Model/Input	Cohort and Validation	Inference Level/Reference Standard	Segmentation	Main Translational Message
Li et al., 2023 [30]	T2WI deep learning compared with VI-RADS readers	Two-center retrospective cohort; internal and external testing	Tumor-level classification; pathology-based muscle invasion label	Manual ROI selection	Strong proof of concept; most relevant gain in equivocal tumors, but workflow still depends on curated lesion selection
Li et al., 2023 [31]	Radiomics vs. single-task DL vs. multi-task DL on MRI	Retrospective comparison with external cohort	Tumor-level classification; pathology-based label	Segmentation-dependent	Architecture matters; multi-task learning outperformed simpler radiomics and single-task models
Kurata et al., 2024 [33]	Vision transformer on MRI	External test cohort with reader comparison	Tumor-level classification; pathology-based label	Manual ROI and semi-automated ROI tested	Performance was comparable to junior readers but fell when ROI definition was less controlled
Cai et al., 2025 [32]	Multicenter T2WI deep learning	Development, validation, internal test, and external test cohorts	Patient/tumor classification depending on dataset; pathology-based label	Not fully end-to-end	External degradation highlighted persistent domain shift despite multicenter design
Cai et al., 2025 [34]	MRI + radiomics + morphology + clinical multimodal fusion	1131 patients from eight institutions; external testing	Patient-level overall survival modeling	Mixed manual and engineered inputs	High prognostic discrimination, but still exploratory and vulnerable to retrospective confounding
Yu et al., 2024 [41]	MRI-based nomogram focused on VI-RADS 3	Retrospective development and validation study	Lesion/tumor-level reclassification within the gray zone	Region-based features	Illustrates how clinically targeted tools may matter more than global AUC improvements
Ye et al., 2023 [46]; Moribata et al., 2023 [47]	Segmentation-focused radiomics and CNN workflows	Two-center or retrospective methodological studies	Pipeline-enabling rather than final pathway studies	Manual vs. semi-automated vs. automated compared	Segmentation burden remains a practical determinant of scalability and reproducibility
Cao et al., 2024 [25]	Public multicenter dataset with federated-learning baseline	Four centers; shared benchmark resource	Dataset and infrastructure resource	Pixel-level annotations provided	Important step toward reproducible multicenter development and privacy-preserving collaboration, but not itself a deployable clinical tool

**Table 4:** Major implementation barriers and which mitigation strategies are available now versus still infrastructure dependent.

Barrier	Why It Matters	What Is Realistically Available Now	What Still Requires Major Development
Acquisition heterogeneity and domain shift	Performance may collapse across scanners, distension states, post-biopsy timing, or site-specific protocols.	Protocol checklists, image-quality gates, site-held-out testing, and local recalibration.	Large harmonized multicenter networks and durable cross-vendor generalization.
Imperfect reference standard	TURBT understaging can contaminate training labels and distort validation.	Explicit reporting of label source, sensitivity analyses, and use of cystectomy or longitudinal labels when available.	Prospective paired imaging-pathology programs and richer lesion-to-pathology matching.
Lesion-level versus patient-level mismatch	High lesion AUC does not necessarily improve the actual treatment decision.	Clear declaration of unit of analysis and patient-level secondary analyses.	Prospective MDT studies linking AI output to management change and outcomes.
Manual segmentation burden	Manual contours reduce scalability and introduce operator variability.	Semi-automated tools, contour QA, and targeted use in equivocal cases.	Reliable end-to-end detection and classification in routine clinical workflows.
Poor calibration and threshold definition	A strong AUC can still produce unsafe recommendations at clinically relevant cutoffs.	Calibration plots, uncertainty reporting, and pre-specified decision thresholds.	Thresholds prospectively validated against treatment pathways and patient outcomes.
Limited interpretability	Saliency overlays rarely provide management-grade explanations.	False-case review, segmentation overlays, and radiologist-facing confidence outputs.	Mechanistically grounded explanations linked to pathology, biology, and failure modes.
Software, governance, and workflow ownership	Even accurate models fail if they are not integrated, auditable, or assigned to a responsible user.	PACS-linked pilot deployment, local governance, radiologist sign-off, and audit trails.	Scaled multi-site implementation of science and reimbursement models.
Limited utility and cost-effectiveness evidence	Adoption depends on changed management and acceptable resource use, not only technical performance.	Pilot pathway studies and time-to-treatment endpoints.	Randomized or pragmatic prospective utility studies with patient-centered outcomes and economic evaluation.

## Data Availability

No new data were created or analyzed in this study.
